# Experimental Investigation of Fabricated Graphene Nanoplates/Polystyrene Nanofibrous Membrane for DCMD

**DOI:** 10.3390/polym13203499

**Published:** 2021-10-12

**Authors:** Ahmad Abdullah, Abdulaziz Al-Qahatani, Mohammed Alquraish, Colin Baily, Salah El-Mofty, Ahmed El-Shazly

**Affiliations:** 1 Department of Civil Engineering, College of Engineering, University of Bisha, P.O. Box 551, Bisha 67614, Saudi Arabia; selmofty@ub.edu.sa; 2 Department of Civil Engineering, Faculty of Engineering, Aswan University, Aswan 81542, Egypt; 3 Department of Biology, College of Science, University of Bisha, P.O. Box 551, Bisha 61922, Saudi Arabia; arabe@ub.edu.sa; 4 Department of Mechanical Engineering, College of Engineering, University of Bisha, P.O. Box 551, Bisha 67614, Saudi Arabia; malqraish@ub.edu.sa; 5 School of Engineering and Materials Science, Queen Mary University of London, Mile End Road, London E1 4NS, UK; principal@qmul.ac.uk; 6 Chemical and Petrochemicals Engineering Department, Egypt-Japan University of Science and Technology (E-JUST), Alexandria 21934, Egypt; elshazly_a@yahoo.com

**Keywords:** membrane distillation, ftir, graphene nanoplates, membrane fabrication, electrospinning

## Abstract

In recent decades, the fabrication of composite membranes using nanoparticles has played a vital role in membrane distillation (MD) technique. It could make available membranes with superior characteristics as excellent candidates for MD technique. The most well-known obstacles regarding the MD method are the low productivity and high cost. Thus, fabricating membranes with superior properties is a significant challenge. In the current study, a composite membrane has been fabricated using 0.25, 0.5, and 0.75 weight percent (wt.%) of graphene nanoparticles (GNPs) with polystyrene (PS) as a base polymer and characterized using SEM, FTIR, and contact angle. The characterization results prove the successful fabrication using electrospinning and the validity of the fabricated membranes to be applied to direct contact membrane distillation (DCMD). In addition, a DCMD experimental setup has been designed to examine the performance of the fabricated membranes and compare the performance of blank PS with composite PS/GNPs membranes. The results show that all fabricated membranes produced an approximately similar average flux of about 10 kg/m^2^ h, while the highest GNPs wt.% showed the highest salt rejection. Accordingly, this composite membrane has been examined at different operating parameters and showed stable performance. Moreover, feed temperature and the rate of flow have a positive impact on the overall performance of the DCMD.

## 1. Introduction

**Figure 1:**
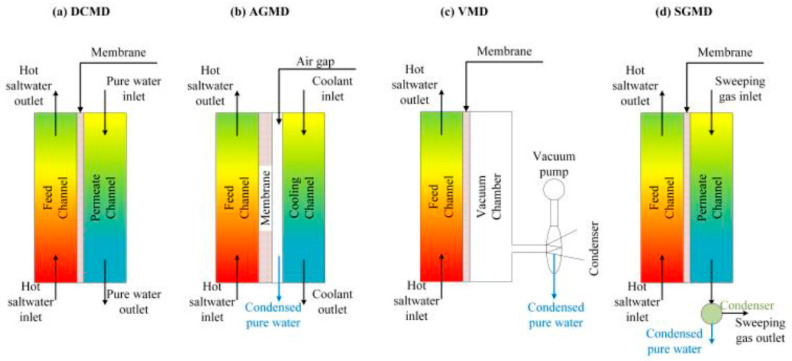
The major types of membrane distillation (**a**) DCMD, (**b**) AGMD, (**c**) VMD, and (**d**) SGMD.

Composite membranes are now playing a vital role in the area of water purification. The stand-out feature of these kinds of membranes is the possibility of changing the fillers, which may be metal-organic frameworks (MOFs), or just controlling the weight percent of a certain MOF to obtain a different enhanced property. Furthermore, it could reduce the fabrication cost compared to available commercial membranes [1]. One of the most critical applications is the membrane distillation (MD) technique, which acts on a thermal basis, and shows good performance and excellent rejection rate [2]. The operating mechanism of MD depends on the vapor transfer across the utilized hydrophobic membrane due to the difference in vapor pressure across the membrane surfaces, which is a consequence of the temperature difference on both sides of the membrane [3,4,5]. There are four major types of MD: (1) direct contact membrane distillation (DCMD), (2) membrane distillation with air gap (AGMD), (3) membrane distillation using sweeping gas (SGMD), and (4) a vacuum based membrane distillation (VMD) [6,7,8]. Figure 1 shows a schematic diagram that illustrates the mechanism of the four mentioned types. In DCMD, the hot and cold water flow across the membrane surfaces provides a vapor pressure difference that forces the vapor to be transferred through the membrane pores to be condensed in the permeate channel. In AGMD, an additional air gap is supplied to reduce the heat transfer and then improves the system thermal efficiency. Furthermore, in VMD, a vacuum is applied behind the membrane which allows more vapor to be transferred and increase the system productivity. Finally, in SGMD, an inert gas is used to remove the vapor and then flow through a condenser to obtain the pure water. Among the four types, DCMD is the most stable and provides a satisfactory performance, due to its simple construction and reduced accessories [9]. It is composed of two channels, one for the salt hot water flow, the second for the cold pure water flow and the in-between hydrophobic porous membrane [10]. There are two configurations based on flow direction: (1) parallel flow and (2) counterflow [11,12]. 

Various polymers are available for membrane fabrication, especially polystyrene (PS), which has proven to be economical and has superior characteristics regarding spinnability, and produces a hydrophobic membrane with suitable porosity for the MD process [13]. Electrospinning is a technique used for providing fibers with nano-sized diameters with excellent porosity, so it is an ideal candidate for MD membrane fabrication [14,15]. The morphology and characteristics of the electrospun fabricated membranes depend on the polymer solution conditions, the surrounding atmosphere, and the setting of the electrospinning machine [16,17,18]. 

One of the most recent fillers added to the polymers-based solution to fabricate a composite membrane with superior characteristics are MOFs, which are suitable for MD [19]. GNPs have also attracted the attention of researchers recently for its hydrophobic character, thermal and mechanical stability, and ion selectivity. Hence adding GNPs to MD membranes was found to boost some membrane characteristics, such as antifouling and increasing the tortuosity of the fabricated membranes for water pathways [20,21]. In the last decade, much research has been conducted on fabricating novel membranes suitable for water purification. An electrospun fabricated polyvinylidene fluoride (PVDF) membrane has been fabricated successfully with superior properties [22]. The authors concluded that the fabricated PVDF membrane has a mostly steady and stable operation on DCMD for 15 h of continuous operation. They also found that the system productivity of pure water when applying the fabricated PVDF membrane on the DCMD system was 21 kg/m^2^ h, at operating conditions of 20 °C, 50 °C, and 35,000 ppm for cold water temperature, hot saltwater temperature, and feed salinity, respectively. Another approach introduced a different PVDF membrane fabrication using a facile bottom-up method with a predicted pure water production of 41.4 kg/m^2^ h when operating at cold and hot side temperatures of 20 °C and 70 °C, respectively [23]. The authors also concluded that the low cost of the proposed technique allowed it to be a superior candidate for large-scale production. 

A numerical simulation was introduced to compare the performance of a fabricated blank PS membrane with a fabricated multiwalled carbon nanotubes composite (MWCNTs) on DCMD [24]. They used the electrospinning technique for the membrane fabrication, proved the successful fabrication via SEM characterization, and showed that the fabricated membrane has a hydrophobic nature by measuring the surface contact angle. The authors found that the composite membrane outperforms the blank membrane at any operating conditions. Such performance was attributed to the improved porosity, which was about 28%. A novel membrane was fabricated using the electro-blowing method to obtain fibers with nanoscale, forming a composite membrane of styrene-acrylonitrile in N, N-dimethylformamide (DMF) [25]. The authors checked the fabricated membrane via SEM and show the validity of the proposed technique. They also studied the fabrication conditions and introduced their effect on the membrane’s surface morphology, which was captured by contact angle and liquid entry pressure tests. They found that the performance on the DCMD system was promising, however much thinner or thicker the utilized membrane was. A super-hydrophobic membrane labeled (FZP) was fabricated to be applied with MD technologies [26]. The authors reported an excellent contact angle of 162.3° for the electrospun fabricated membrane and higher LEP. They concluded that their proposed fabricated membrane could fix the anti-wetting problems that are related to other membranes.

On the other hand, a mechanical vision for improving the overall performance of the MD technique could be achieved by improving the heat transfer process and mass transfer. Spacers-filled channels considerably enhance thermal performance and reduce the temperature gradient by promoting turbulences [27,28]. The most recent techniques used to overcome the polarization in temperature challenge in MD were comprehensively reviewed [8]. Additionally, renewable energy (i.e., solar energy) has been hybridized with MD for two purposes: the first one is to produce fresh water and electricity simultaneously, and the second purpose is to build an energy-efficient, economical, and ecofriendly MD system that excludes any external conventional power supply [29,30,31].

A comprehensive review of the polymeric membranes has been conducted to highlight the importance of the newly fabricated composite membrane in the desalination and purification field [32]. The authors reported the new trend of many researchers to use a renewable polymer to reduce the number of technical polymers. A hybrid filtration to extract mercury (II) from water has been introduced [33]. They characterized the produced water with time to examine the water quality. They also concluded that the membrane could be successfully reused without further chemical processing. On the other hand, Polysulfone, which is considered one of the most important polymers for membrane fabrication, has been concisely reviewed [34]. The authors reported the superior properties of that polymers, such as mechanical and thermal stability, which recommended it as an excellent candidate for membrane fabrication. Additional applications have been further introduced in the latest mentioned review, such as catalyst and ion exchange. An interesting modification has been performed on a nanofiltration membrane by adding modified graphene oxide, that was applied on water desalination [35]. The modified membranes have been completely characterized using SEM, TEM, FTIR, etc., to ensure their successful preparation. As a result, the modified membrane was significantly outperforming the basic membrane.

In this regard and following the available literature, the main obstacle of the MD technique is to fabricate a porous membrane with a hydrophobic nature, and excellent characteristics. Using GNPs (0.25 wt.%) with PS to fabricate a composite membrane has been introduced in the authors’ recent published work [36]. It was investigated numerically to study the effect of operating conditions on the system productivity, coefficient of polarization, and efficiency. In the current work, the authors aim to study the effect of the weight percent of GNPs on the fabricated membranes. In addition, the present work introduces the experimental results of the fabricated membranes when used in the DCMD system. Also, it experimentally introduces the stability of the fabricated membranes with time and compares the quality of the produced fresh water. The main target of any new fabricated membrane is to provide high freshwater production and high salt rejection. Therefore, the originality of the proposed study is to composite polystyrene (PS) with graphene nanoplates (GNPs) to fabricate composite PS/GNPs membranes for the MD process. The fabrication process was performed via electrospinning using different weight percentages (wt.%) to compare the composite membrane with the blank PS performance on DCMD. The DCMD used is a lab-scale test rig that compares composite PS/GNPs with blank PS at different operating conditions.

## 2. Materials and Methods

Polystyrene (PS) (pellets, Mw = 192,000) purchased from Alpha Chemika, Mumbai, India; and N,N-dimethyl formamide (DMF) (99.8% GC, ACS reagent) and Graphene nanoplatelets (GNPs) (Carbon > 85 wt.%) from Sigma-Aldrich, Taufkirchen, Germany.

### 2.1. Fabrication Process

The first step is to dissolve PS in DMF, followed by 6 h of stirring at normal room conditions. The weight percent followed of PS was the optimum value (18 wt.%) that has been concluded by [4]. Authors of that study used PS solution concentration of (15, 18, and 20 wt.%) for electrospinning at fixed voltage, flowrate and tip to collector distance –mentioned later in this paragraph – and concluded that 18 wt.% PS solution gives continuous, uniform beadles fiber. The weight percentages of GNPs introduced in the current work were (0.25, 0.5, and 0.75) wt.%, added to the prepared polymer solution. The composite solution was left in sonication for 1 h after a similar stirring period to assure a homogeneous solution. After that, the electrospinning was set for: (a) a needle discharge of 0.6 mL/h, (b) a distance between the tip and collector of 18 cm, and (c) an applied voltage difference of 28 kV. Finally, the proposed samples were kept overnight in the oven at 60 °C, then pressed via cold press under 2000 kPa for 1 min.

### 2.2. Characterization

Characterization for the fabricated membranes is mandatory to ensure the successful fabrication of blank and composite membranes and the validity of the fabricated membrane for the target application. Therefore, the following characterizations were applied in the current work:

#### 2.2.1. Scanning Electron Microscope (SEM)

The surface morphology of the produced membranes was studied using (JSM-6010 LV SEM, Tokyo, Japan). Samples of the blank and composite membranes were fixed on an aluminum stub using carbon tape and vacuumed for two minutes under an accelerating voltage of 20 mV.

#### 2.2.2. Fourier-Transform Infra-Red (FTIR)

Pellets of the blank and composite membranes samples were formed by mixing with KBr in the ratio of 1:100 *w/w* and then studied using FTIR (Bruker scientific instruments, Vertex 70, Baden Württemberg, Germany) at room temperature. The wave range used was 4000–400 cm^−1^.

#### 2.2.3. Contact Angle

A static water contact angle test was carried out for the fabricated membranes to study the surface hydrophobicity. In this test, a single drop of water is dropped onto the membrane surface and captured by a high-speed camera at the drop-surface interface. A drop size analyzer (DSA 100, Kruss), paired with software to analyze the image, measured the water’s contact angle, and an average value of measurements at ten different spots was taken.

## 3. Experimental Work

**Figure 2:**
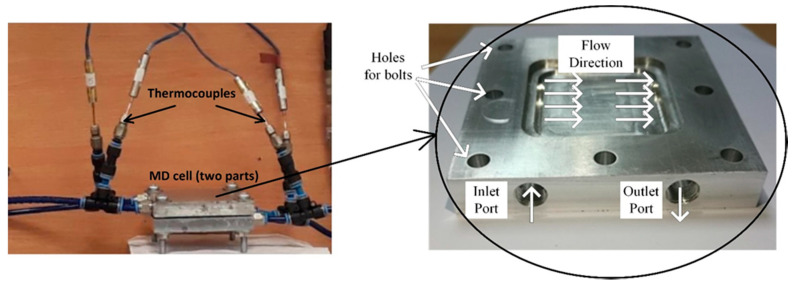
Experimental membrane distillation test rig.

The fabricated (blank PS and composite PS/GNPs) membranes’ performance was examined on DCMD existing system. The system is composed of two identical aluminum parts representing the hot and cold channels. The fabricated sample (membrane) was fixed between the channels with the addition of gaskets to prevent any leakages. Figure 2 shows the existing MD system, as can be seen from the figure, the two parts were adjusted together, and the thermocouples were fitted at the inlet and outlet ports of both hot and cold flow channels. These thermocouples were connected to a data logger to capture the temperature at the recommended points digitally. The left figure shows a magnification for the flow channel.

Table 1 shows the system dimensions. Two pumps were used to feed the system with fluids, one for the pure water and the other for the saltwater. The used pumps are suitable for the low flow rate range to provide the recommended flow rate, i.e., to be within the laminar flow regime.

The ranges of the operating parameters that were used in the current work are listed in Table 2. As detailed in the table, the present study was performed at different operating conditions (i.e., saltwater temperature, flow rate, and feed salt concentration) and various weight percentages of GNPs, to examine the membrane performance at different conditions.

After utilizing the fabricated membranes, the temperatures at the inlet and outlet for cold and hot water were captured through the data logger. Furthermore, the flow rate was indicated on the pumps monitors and could be adjusted manually to control the pumps’ discharge. The salt concentration of the feed was measured at the feed inlet and the permeate outlet to highlight the rejection rate of the proposed membranes when applied to the DCMD system.

## 4. Results and Discussion

The results regarding the proposed experimental work have two main issues. One is relevant to the fabricated membranes’ characterization, which introduces the evidence of the successful fabrication and existence of the proposed nanoparticles in the polymer solution. Moreover, some of these characterizations show the validity of the fabricated membranes to be applied to the MD technique. On the other hand, the second part of the results shows the performance of the fabricated membranes when applied to the DCMD system at different operating conditions. The actual performance test on the DCMD system examines the fabricated membranes at different saltwater temperatures, flow rates, and feed salinity.

### 4.1. Membranes’ Characterization

**Figure 3:**
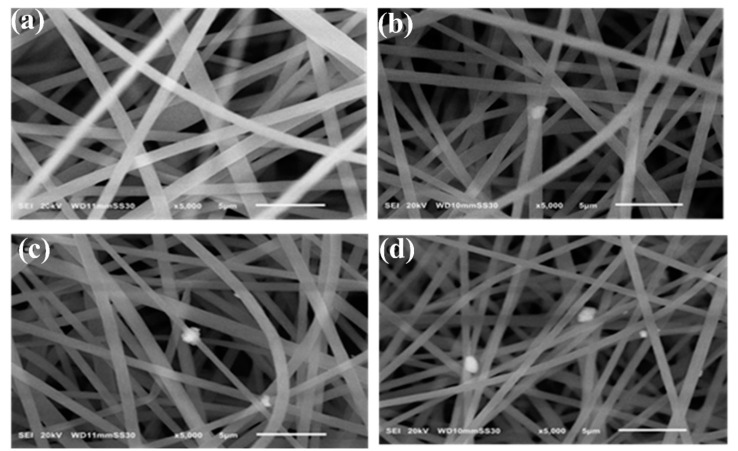
SEM images: (**a**) Blank PS membrane (**b**) Composite PS/GNPs membrane with 0.25 wt.% (**c**) Composite PS/GNPs membrane with 0.5 wt.% (**d**) Composite PS/GNPs membrane with 0.75 wt.%.

Figure 3 displays the SEM views of neat PS and composite PS/GNPs samples at the given electrospinning conditions. The produced fibers’ uniform and beadles feature ensures that the selected electrospinning conditions are adequate for the proposed PS solution. The average fiber diameter of the blank PS membrane was 1.013 μm, whereas that of the composite fiber was 0.719 μm. This decrease in the average fiber diameter is a consequence of the enhanced conductivity of the PS doped solution due to the addition of GNPs [20]. Increasing the GNPs concentration also resulted in some aggregate formation as can be noticed in Figure 3c,d this observation is in agreement with [20].

**Figure 4:**
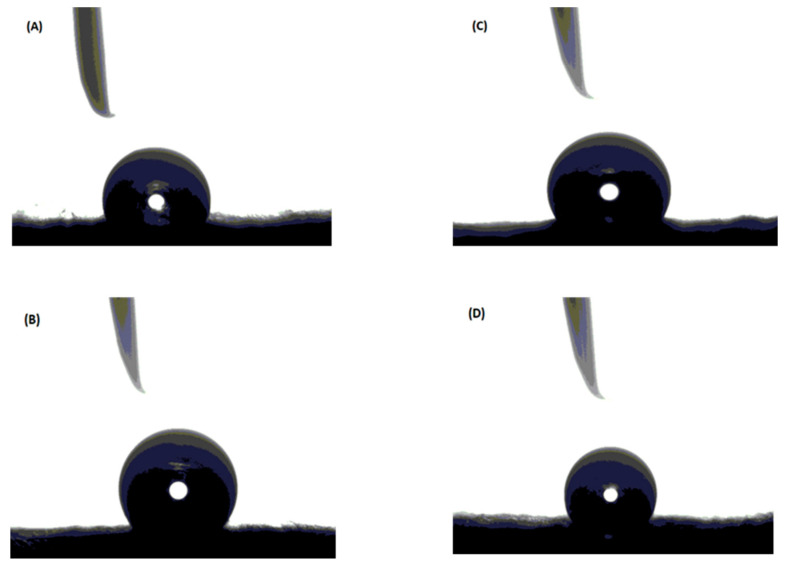
Contact angle for: (**A**) Blank PS membrane (**B**) Composite PS/GNPs membrane with 0.25 wt.% (**C**) Composite PS/GNPs membrane with 0.5 wt.% (**D**) Composite PS/GNPs membrane with 0.75 wt.%.

The addition of GNPs also significantly enhanced the fabricated membranes’ hydrophobicity. The static water contact angle increased from 73.79° for the blank PS membrane to 91.68°, 95.65°and 105.08° for the PS/GNPs with 0.25 wt.%, 0.5 wt.%, and 0.75 wt.%, respectively, as shown in Figure 4. This increase in membrane hydrophobicity may be attributed to the increased surface roughness as the concentration of the added GNPs increases [24].

**Figure 5:**
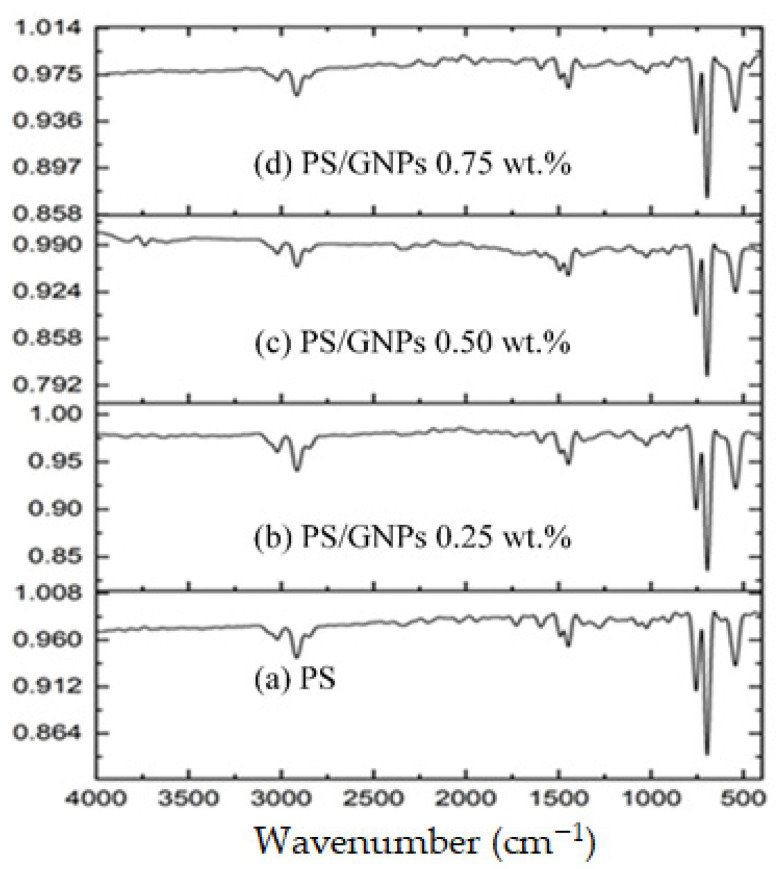
FTIR for: (**a**) Blank PS membrane (**b**) Composite PS/GNPs membrane with 0.25 wt.% (**c**) Composite PS/GNPs membrane with 0.5 wt.% (**d**) Composite PS/GNPs membrane with 0.75 wt.%.

The IR spectra of the fabricated neat PS and composite PS/GNPs membranes are given in Figure 5. The symmetric and asymmetric vibration of the C-H group is observed from 3100–2800 cm^−1^ in all the IR spectra. The pronounced peaks in the range of 2000–1680 cm^−1^ are due to the aromatic mono substitution, while the peaks observed at 1446.41 cm^−1^ are attributed to the CH_2_ bending vibration. The characteristic peaks at 754.61 and 695.93 and 543 cm^−1^ are assigned to the phenyl ring’s CH out-of-plane bending vibrations and CH out-of-plane deformation, respectively [37,38]. These peaks confirms that PS structure was not changed and that no chemical reactions took place during the compositing process to alter the structure of PS. The effect of the addition of GNPs is pronounced in increasing the strength of the characteristic peaks by increasing the percentage of GNPs added [39].

### 4.2. Membranes’ Performance

In this section, the performance of the fabricated composite PS/GNPs membranes would be introduced at different operating conditions and would be compared with the blank PS membrane. At first, the performance of all fabricated membranes (i.e., blank PS, 0.25 wt.% composite PS/GNPs, 0.5 wt.% composite PS/GNPs, and 0.75 wt.% composite PS/GNPs) would be compared. The comparison of these membranes would concern the productivity and the quality of the produced water (i.e., total dissolved salts (TDS)) when operated at the same conditions for a continuous period of 7 h. After that, the membrane which recorded the best performance would be examined separately at different operating conditions to highlight the effect of the mentioned operating parameters.

**Figure 6:**
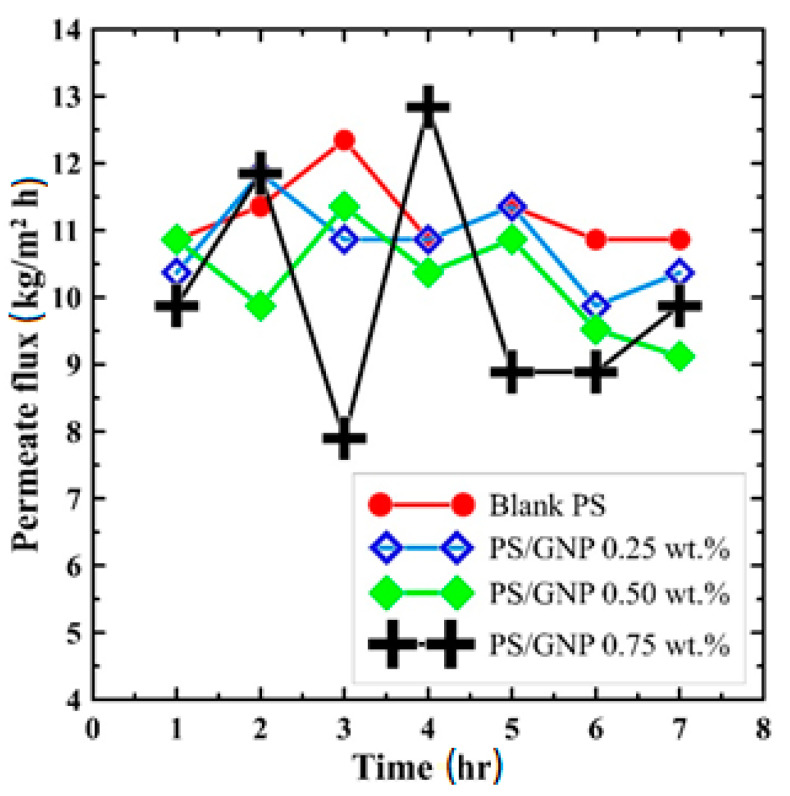
Comparison of permeate flux for all fabricated membranes over 7 h operating time.

At the outset, an experimental comparison was carried out on the performance of the fabricated membranes in the DCMD at constant operating conditions. The saltwater feed temperature and the cold pure water temperature were adjusted to fixed values of 64 °C and 12 °C, respectively. The water flow rate was kept at 100 mL/min, while the salinity of the hot water was adjusted to 10,000 ppm. Figure 6 shows the productivity of blank PS, composite PS/GNPs with (0.25, 0.5, and 0.75) wt.% of GNPs over the operating time of 7 h. The figure shows that the fabricated membrane has a largely similar productivity which fluctuated around an average value of almost 10 kg/m^2^ h. This fluctuation may be attributed to the uncertainty in measurements, although this trend is similar to that reported in [40]. One could conclude from this figure that adding GNPs to the blank PS would not significantly affect the system productivity.

**Figure 7:**
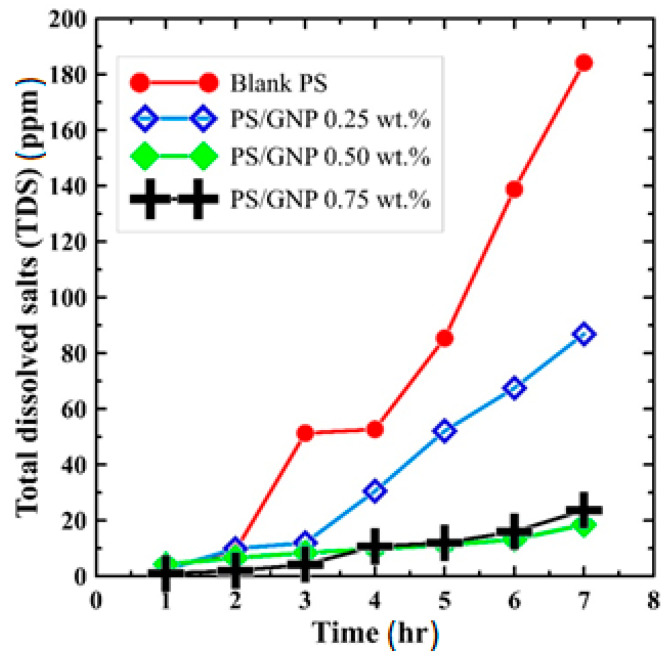
Comparison of permeate flux quality (TDS) for all fabricated membranes over 7 h operating time.

Indeed, as can be seen in Figure 7, blank PS has the worst permeate water quality, which increases dramatically with time. After one hour of operation, the produced pure water quality was about 2.5 ppm, and after the test period, the water quality reached a value of 185 ppm. On the other hand, all the composite PS/GNPs membranes have considerably higher quality (lower TDS). However, the composite PS/GNPs membranes with 0.5 wt.% and 0.75 wt.% have the best quality, which is approximately similar, noting that the 0.75 wt.% composite has slightly higher quality. For instance, in the first three hours, the composite membranes produced water of nearly similar quality. After 3 h, the quality of the produced water from the 0.25 wt.% composite PS/GNPs membrane has considerably decreased as it recorded TDS of about 85 ppm after 7 h. The other 0.5 and 0.75 wt.% composite PS/GNPs membranes have an excellent quality after the test period, as the maximum recorded TDS was about 20 ppm. Accordingly, the composite PS/GNPs membrane of 0.75 wt.% has the best performance amongst the whole studied membranes on the DCMD system, of about 10 kg/m^2^ h productivity and 99.8% salt rejection.

Accordingly, the 0.75 wt.% composite PS/GNPs membrane has been selected to be a candidate for the DCMD system. Consequently, this membrane has been tested on DCMD to study its performance at different operating conditions. The targeted performance parameters are the produced pure water (permeate flux) and the quality of the produced water (i.e., TDS) as these are the most important criteria for membrane distillation. For the subsequent results, the experimental runs were repeated four times to obtain the uncertainty, and this is presented on the following figures as error bars. 

**Figure 8:**
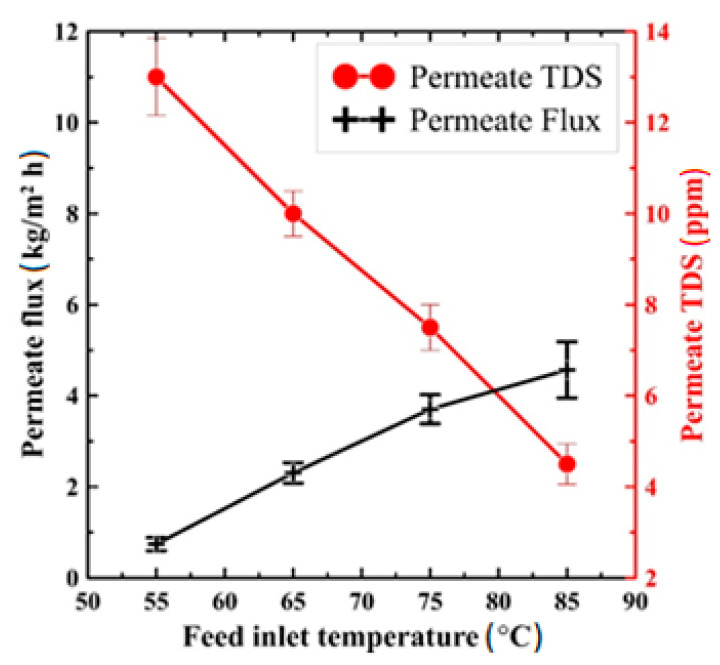
Effect of feed inlet temperature on permeate flux and TDS of the produced water at 100 mL/min flow rate and 10,000 ppm feed salt concentration.

Figure 8 shows the effect of hot saltwater temperature on the permeate flux and produced water TDS at 100 mL/min rate of flow and 10,000 ppm feed salt concentration. According to the actual performance on DCMD presented in Figure 8, the composite PS/GNPs membrane proved a stable operation with good productivity of about 4.5 kg/m^2^ h at the maximum studied temperature and excellent water quality of 4.5 ppm (i.e., 99.955% salt rejection). It is also very clear from the figure that the feed inlet temperature considerably impacts the overall performance. For example, increasing the temperature from 55 °C to 85 °C increased the productivity from 0.75 kg/m^2^ h to 4.5 kg/m^2^ h, with the water TDS being reduced from 13 ppm to 4.5 ppm, respectively. This finding matches with that concluded in [41,42,43]. This improvement due to increased temperature is attributed to the increased vapor pressure difference (vapor transfer driving force). It is a direct consequence of the temperature difference across the membrane surfaces and hence enhanced productivity.

**Figure 9:**
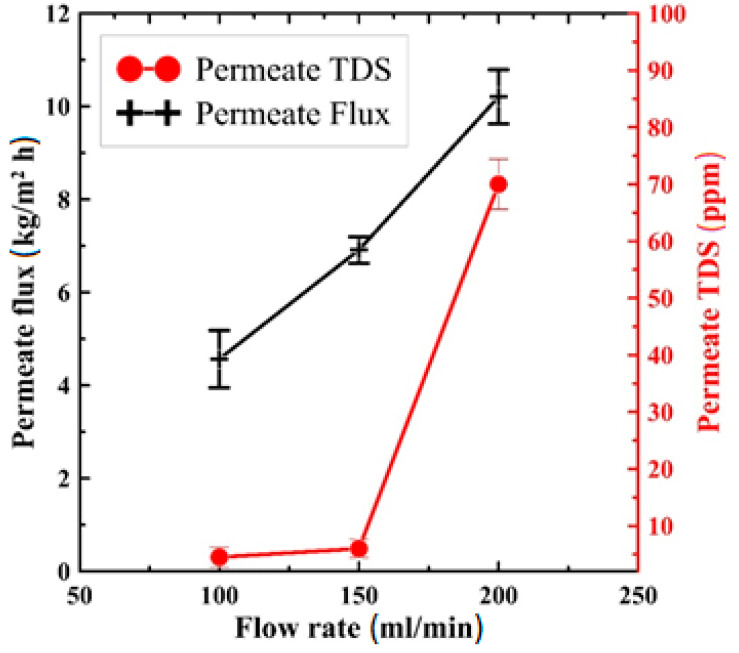
Effect of feed flow rate on permeate flux and TDS of the produced water at 85 °C saltwater inlet temperature and 10,000 ppm feed salt concentration.

Furthermore, the effect of flow rate on productivity and quality of the produced water has been presented in Figure 9. This experimental test has been carried out at the maximum mentioned temperature (85 °C) and fed at a salt concentration of 10,000 ppm. As shown in Figure 9, increasing the rate of flow improves the system productivity considerably, but it negatively affects the quality of the produced water. As presented in the figure, increasing the rate from 100 to 200 mL/min increases the produced pure water flux from 4.5 kg/m^2^ h to about 10 kg/m^2^ h which is significantly higher than that produced from [44], while the produced pure water salinity was increased (negative effect) from 4.5 ppm to 70 ppm. The physical concept here is that increasing the flow rate would improve the heat transfer coefficient in the two-fluid parts, decreasing the temperature polarization phenomenon. This will keep the membrane surfaces’ temperature as close as possible to the fluid bulk temperature, increasing the driving force and system productivity. This result matches well with that introduced in [9,41,45].

**Figure 10:**
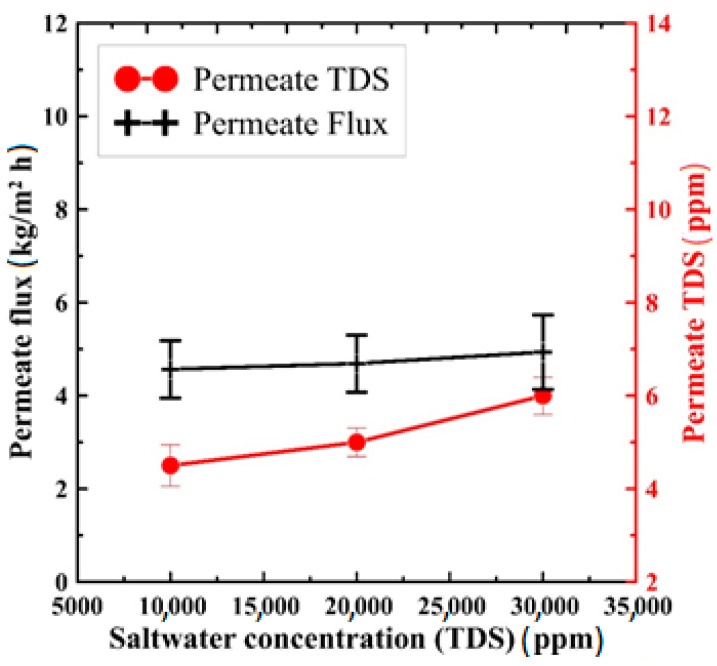
Effect of feed salt concentration on permeate flux and TDS of the produced water at 85 °C saltwater temperature and 100 mL/min rate of flow.

Finally, the effect of feed salt concentration on both system productivity and the pure water quality has been introduced in Figure 10. This result has been conducted at 100 mL/min and 85 °C, rate of flow, and saltwater temperature, respectively. At the same time, the permeate was kept at a fixed temperature of 12 °C. As can be seen in the figure, increasing the saltwater concentration within the studied range shows a negligible effect on the produced permeate flux, as increasing the feed TDS from 10,000 ppm to 30,000 ppm shows a productivity change of about 5%. On the other hand, increasing the feed salinity reduces the produced water TDS. As shown in the figure, increasing the feed TDS from 10,000 ppm to 30,000 ppm has increased the TDS of the produced water from 4.5 ppm to 6 ppm, which means that the salt rejection has been improved from 99.955% at 10,000 ppm to be 99.98% at 30,000 ppm. This improved rejection rate is a direct consequence of the nature of the fabricated PS/GNPs sample, as GNPs additives (0.75 wt.%) play a vital role in the rejection process if compared to the blank PS membrane, as illustrated in Figure 7.

## 5. Conclusions

The nanoparticles composite membrane has been successfully fabricated using three different weight percentages (wt.%) of 0.25, 0.5, and 0.75 of graphene nanoplates (GNPs) with the base polymer polystyrene (PS). The membranes, fabricated via electrospinning technique have been characterized using SEM, FTIR, and contact angle, present a successful fabrication. The contact angle of the composite membranes was captured and found to be higher than that of the blank membrane. The highest recorded contact angle was 105.08° for the 0.75 wt.% composite PS/GNPs membrane. Utilizing the blank and composite fabricated membranes in the DCMD system shows almost stable operation over the test period of approximately similar productivity of 10 kg/m^2^ h, while the 0.75 wt.% composite PS/GNPs membrane produces pure water with the best quality of about 99.955%. Moreover, this membrane was tested at different operating conditions to check its stability and performance. The experimental results show that this membrane is stable over the test time and under the studied range of operating conditions. It has a productivity of about 4.5 kg/m^2^ h at the proposed feed temperature, lowest mentioned rate of flow, and 10,000 ppm salinity, with a salt rejection of 99.955%.

## Figures and Tables

**Table 1:** System dimensions.

Item	Dimension
Channel length	4.5 cm
Channel width	4.5 cm
Channel height	2.5 mm
Active area	20.25 cm^2^
Inlet port diameter	4 mm
Outlet port diameter	4 mm

**Table 2:** System dimensions.

Item	Dimension
Flow rate	100–200 mL/min
Saltwater inlet temperature	55–85 °C
Coldwater temperature (permeate)	12 °C
Salt concentration	10,000–30,000 ppm
GNPs wt.%	0.25–0.75%

## Data Availability

Not applicable.
